# The Codevelopment of “My Kidneys & Me”: A Digital Self-management Program for People With Chronic Kidney Disease

**DOI:** 10.2196/39657

**Published:** 2022-11-14

**Authors:** Courtney J Lightfoot, Thomas J Wilkinson, Michelle Hadjiconstantinou, Matthew Graham-Brown, Jonathan Barratt, Christopher Brough, James O Burton, Jenny Hainsworth, Vicki Johnson, Maria Martinez, Andrew C Nixon, Victoria Pursey, Sally Schreder, Noemi Vadaszy, Lucina Wilde, Fiona Willingham, Hannah M L Young, Thomas Yates, Melanie J Davies, Alice C Smith

**Affiliations:** 1 Leicester Kidney Lifestyle Team Department of Health Sciences University of Leicester Leicester United Kingdom; 2 Leicester Biomedical Research Centre Leicester United Kingdom; 3 National Institute for Health Research Applied Research Collaboration East Midlands Leicester Diabetes Centre Leicester United Kingdom; 4 Diabetes Research Centre University of Leicester Leicester United Kingdom; 5 Department of Cardiovascular Sciences University of Leicester Leicester United Kingdom; 6 Department of Renal Medicine University Hospitals of Leicester NHS Trust Leicester United Kingdom; 7 Leicester Diabetes Centre University Hospitals of Leicester NHS Trust Leicester United Kingdom; 8 School of Sport, Exercise and Health Sciences Loughborough University Loughborough United Kingdom; 9 Department of Medical Psychology Leicestershire Partnership NHS Trust Leicester United Kingdom; 10 University Hospitals of Leicester NHS Trust Leicester United Kingdom; 11 Renal and Transplant Unit University Hospitals of Leicester NHS Trust Leicester United Kingdom; 12 Department of Renal Medicine Lancashire Teaching Hospitals NHS Foundation Trust Preston United Kingdom; 13 Division of Cardiovascular Sciences University of Manchester Manchester United Kingdom; 14 Department of Renal Medicine York Teaching Hospital NHS Foundation Trust York United Kingdom; 15 Nutrition and Dietetics Team, School of Sport and Health Sciences University of Central Lancashire Preston United Kingdom; 16 Nutrition and Dietetic Department East Lancashire Hospitals NHS Trust Blackburn United Kingdom; 17 Diabetes Research Centre, College of Life Sciences University of Leicester Leicester United Kingdom

**Keywords:** kidney disease awareness, kidney disease knowledge, program development, eHealth, digital health, telehealth, mobile health, mHealth, health promotion, self-management behaviors, mobile phone

## Abstract

**Background:**

Health care self-management is important for people living with nondialysis chronic kidney disease (CKD). However, the few available resources are of variable quality.

**Objective:**

This work describes the systematic codevelopment of “My Kidneys & Me” (MK&M), a theory-driven and evidence-based digital self-management resource for people with nondialysis CKD, guided by an established process used for the successful development of the diabetes education program MyDESMOND (Diabetes Education and Self-Management for Ongoing and Newly Diagnosed, DESMOND).

**Methods:**

A multidisciplinary steering group comprising kidney health care professionals and researchers and specialists in the development of complex interventions and digital health provided expertise in the clinical and psychosocial aspects of CKD, self-management, digital health, and behavior change. A patient and public involvement group helped identify the needs and priorities of MK&M and co-design the resource. MK&M was developed in 2 sequential phases. Phase 1 involved the codevelopment process of the MK&M resource (content and materials), using Intervention Mapping (IM) as a framework. The first 4 IM steps guided the development process: needs assessment was conducted to describe the context of the intervention; intervention outcomes, performance objectives, and behavioral determinants were identified; theory- and evidence-based change methods and practical strategies to deliver change methods were selected; and program components were developed and refined. Phase 2 involved the adoption and adaptation of the existing MyDESMOND digital platform to suit the MK&M resource.

**Results:**

The needs assessment identified that individuals with CKD have multiple differing needs and that delivering a self-management program digitally would enable accessible, tailored, and interactive information and support. The intended outcomes of MK&M were to improve and maintain effective self-management behaviors, including physical activity and lifestyle, improve knowledge, promote self-care skills, increase self-efficacy, and enhance well-being. This was achieved through the provision of content and materials designed to increase CKD knowledge and patient activation, reduce health risks, manage symptoms, and improve physical function. Theories and behavior change techniques selected include Self-Management Framework, Capability, Opportunity, Motivation Behavior model components of Behaviour Change Wheel and taxonomy of behavior change techniques, Health Action Process Approach Model, Common Sense Model, and Social Cognitive Theory. The program components developed comprised educational and behavior change sessions, health trackers (eg, monitoring blood pressure, symptoms, and exercise), goal-setting features, and forums for social support. The MyDESMOND digital platform represented an ideal existing platform to host MK&M; thus, the MyDESMOND interface and features were adopted and adapted for MK&M.

**Conclusions:**

Applying the IM framework enabled the systematic application of theory, empirical evidence, and practical perspectives in the codevelopment of MK&M content and materials. Adopting and adapting a preexisting platform provided a cost- and time-efficient approach for developing our digital intervention. In the next stage of work, the efficacy of MK&M in increasing patient activation will be tested in a randomized controlled trial.

## Introduction

### Background

Chronic kidney disease (CKD) affects approximately 5% to 7% of people living in the United Kingdom [1]. People with CKD have an increased risk of morbidity and mortality, which is largely attributable to cardiovascular disease [2]. Progressive kidney damage can lead to end-stage kidney disease (ESKD), where kidney replacement therapy (ie, dialysis or transplantation) may be needed [3]. However, most people with reduced kidney function never progress to ESKD but, nevertheless, live with significant poor health and high symptom burden [4]. Many individuals with nondialysis CKD are diagnosed and managed by primary care clinicians with infrequent or no input from kidney specialists in secondary care or other members of the multidisciplinary team (eg, specialist or nonspecialist nurses or allied health professionals) [5]. These individuals are mainly required to self-manage their condition, which involves balancing the medical, emotional, and psychosocial consequences of living with CKD and any additional comorbidities with their day-to-day responsibilities [6,7].

The Chronic Care Model has identified self-management as an essential component in chronic disease management to empower patients to take a more active role in their health [8]. Self-management is a complex set of processes and tasks involving the following: (1) use of an individual’s knowledge (education), skills, and confidence in managing their disease; (2) the ability to identify and access resources and support; and (3) learning to cope and live with the condition, including the impact on an individual’s life and the emotional consequences [9-11]. The goal of effective self-management support is to identify strategies that can help individuals manage their condition(s) while leading full, active, and productive lives. For people with CKD, specific self-management behaviors range from understanding and adhering to medication, health and symptom monitoring, and making lifestyle modifications (eg, increasing physical activity and eating an appropriate diet), which reduce cardiovascular, CKD, and general health risk factors [12].

Effective self-management requires appropriate knowledge, skills, and confidence to manage one’s own health and care; this is termed *patient activation* [13]. However, significant deficiencies in CKD awareness and knowledge among people with CKD have been identified [14-16], with many reporting limited or no understanding of their condition and little or no awareness about how to manage their health [17]. Individuals living with CKD, who are managed in primary care, are often unaware of their diagnosis and the detrimental effect that poor risk factor control (such as hypertension and diabetes) may have on their health [18].

Evidence suggests that primary health care providers infrequently discuss CKD with their patients and that the quality of these discussions is limited [19]. Furthermore, self-management support and education through face-to-face interactions in clinical settings are minimal [6,20-22]. For clinicians, barriers to successful education include lack of time and clinical confidence combined with competing priorities [17]. For people living with CKD, the complex nature of CKD information, limited awareness, health literacy and numeracy combined with the lack of readily available and understandable information and reduced readiness to learn form the greatest challenges [17]. Clearly, innovative approaches to support self-management in people with CKD are urgently required.

Although various self-management interventions have been evaluated, many are not theoretically driven or evidence-based, nor are they informed by patient preferences [22]. Digital health interventions (DHIs) are an efficient and effective method of providing interventions to improve knowledge and self-management behaviors and to actively involve individuals in their care, resulting in improved outcomes for people with long-term conditions [23]. Implementation barriers often associated with face-to-face interventions, such as time and transport, can be addressed using DHIs, which provide more accessible, acceptable, tailored, and interactive information and support [24,25]. When developing evidence- and theory-based DHIs, it is essential that appropriate methods are adopted to ensure effective implementation [26].

Despite DHIs becoming more readily available for people with CKD, few are theory-based, and the development strategies and processes are unclear. Conversely, DHIs in other long-term conditions, such as type 2 diabetes, have appropriately recorded and systematically communicated their development processes. An example is MyDESMOND, which adapted a face-to-face diabetes self-management program (Diabetes Education and Self-Management for Ongoing and Newly Diagnosed, DESMOND) [9] and translated it onto a digital platform [27]. The adaptation and development of MyDESMOND were guided by the intervention mapping (IM) framework.

### Objective

The aim of this paper is to provide an overview of the process used in the systematic development of “My Kidneys & Me” (MK&M), a digital self-management program for people living with nondialysis CKD.

## Methods

### Overview of Predevelopment Work

#### Review of Previous Research Projects

Before the work described in this paper, we reviewed the experiences and learning from our previous research projects, which were conducted between 2016 and 2018 and involved the development and preliminary evaluation of 2 physical activity behavior change interventions for people with CKD.

#### Physical Activity Changing Together

Underpinned by the social cognitive theory [28], we previously developed PACT (Physical Activity Changing Together) [29,30], a group-based program aimed at increasing the levels of physical activity in people with nondialysis CKD via structured education (face-to-face sessions comprising 6 modules and 3 booklets) and self-monitoring strategies (pedometer and action planner) [29,30]. In a small uncontrolled evaluation, the PACT program was deemed feasible and acceptable to the participants, and the group-based and interactive delivery was well received by them. However, recruitment was poor (recruitment rate of 23%) [30], suggesting that recruiting to a similar intervention in practice may not be viable. Participants suggested refinements to PACT, which predominantly included broadening the focus to provide a more holistic approach to lifestyle management, including dietary information, emotional management, and other types of exercise.

#### Self-directed Programme to Increase Physical Activity in CKD

Underpinned by the theory of planned behavior [31], SPARK (Self-directed Programme to Increase Physical Activity in Chronic Kidney Disease) [32,33] was a one-to-one, self-directed physical activity intervention designed to increase physical activity levels in nondialysis CKD [32,33]. SPARK was an 8-week walking and strength training program that was delivered using one-to-one motivational interviewing sessions, with supportive written educational material (two booklets), biweekly telephone calls, and self-monitoring strategies (pedometers and exercise diary). Initial testing, in a small uncontrolled trial, showed that SPARK was feasible and acceptable, with high levels of engagement and completion of the program [33]. Findings suggested positive changes in physical activity behavior, functional ability, symptom burden, disease knowledge, confidence to exercise, patient activation, and quality of life (QoL). However, similar to PACT, recruitment to the program was poor (recruitment rate of 27% from secondary care and 5% from primary care). Although the completion of the physical activity diary was high, interviews with participants highlighted that strength training was perceived as less important and that they needed improved guidance regarding technique.

#### Learning to Take Forward

Although PACT and SPARK increased disease knowledge and exercise behaviors, there was a clear need for a more holistic approach to support health and lifestyle management, with more guidance on how to engage with and perform desired behaviors. Recruitment to the PACT and SPARK programs was low, and the need to attend the hospital or clinic for face-to-face sessions was cited as a major barrier to participation and engagement. The staff time commitment involved in delivering sessions and follow-up telephone calls also significantly reduced the likely practicality and feasibility of both interventions outside a research setting. Therefore, the overall learning indicated that an alternative method may be needed to deliver the required knowledge and skills, which (1) provides a holistic approach, (2) is likely to be cost-effective, and (3) reduces the number of visits to the clinic or hospital.

### Development of MK&M

#### Overview

There were 2 sequential phases in the development of MK&M:

1. Phase 1: development process of the MK&M resource (content and materials) using IM as a framework (March 2019 to April 2020).
2. Phase 2: adoption and adaptation of the existing MyDESMOND platform to suit the MK&M content and materials (March 2020 to April 2021).

The *Methods* section describes the process and steps of the development work conducted in phases 1 and 2, and the *Results* section presents the details of the needs assessment and theories identified and selected phases 1 and 2.

#### Steering Group

Prior to embarking on Phase 1, a steering group comprising of kidney researchers from the Leicester Kidney Lifestyle team, representatives of the multidisciplinary kidney health care team (including doctors, nurse, pharmacist, dietician, physiotherapist, psychologist), and experts from the Leicester Diabetes Centre with experience in developing complex interventions (including members of the Innovative Management, Prevention and Care for long term Conditions [IMPACT] team) and digital health (including those who were involved in the development of the digital-based self-management programme for type 2 diabetes, MyDESMOND[27]) was convened. The patient and caregiver perspectives were provided by a patient and public involvement (PPI) group comprising 10 (83%) people with CKD (aged between 33 and 78 years; 7/10, 70% female; and 9/10, 90% White British) and 2 (17%) spouses or relatives. The multidisciplinary steering and PPI groups worked together to identify the needs and priorities for a self-management intervention for people with CKD, develop the program content and materials, and design program features. Both groups met regularly to provide input into the development of MK&M throughout phases 1 and 2.

### Phase 1: Codevelopment Process of MK&M Using IM

#### Overview

IM is a framework designed to systematically develop effective theory- and evidence-based behavior change interventions for real-world implementation [34]. IM was created for, and is widely used in, health promotion but can be applied to any situation in which behavior change is desirable [35]. IM has previously been used in the development of rehabilitation and self-management interventions for people with chronic diseases [36], including nondialysis CKD [37,38], kidney transplantation [39], and type 2 diabetes [27]. This approach provides an iterative, six-step decision-making framework for effective intervention planning, implementation, and evaluation. The creation of MK&M encompassed steps 1 to 4 of the IM process: (1) conduct needs assessment to create a logic model of the problem; (2) identify intervention outcomes, performance objectives, behavioral determinants, and construct matrices of change objectives; (3) select theory-informed intervention methods and practical strategies to deliver change; and (4) intervention development, including the drafting and refining of materials. A subsequent clinical trial (SMILE-K [Self-Management Intervention through Lifestyle Education for Kidney health]; ISRCTN18314195) will address the final 2 stages of IM steps: (5) adoption and implementation of the intervention and (6) evaluation and feasibility testing. The IM approach used was similar to and guided by that used in the development of MyDESMOND [27] but was followed separately and driven and informed by CKD experts and PPI representatives who co-designed MK&M, with advice and support from the MyDESMOND team.

#### Step 1: Needs Assessment

##### Overview

The aim of step 1 was to conduct an assessment of the health problem, its related behaviors, and the environmental determinants of the behavior. The needs assessment comprised 4 elements: (1) a literature review of the existing self-management interventions in nondialysis CKD; (2) a previous observational study conducted by our group; (3) a PPI CKD priority-setting workshop; and (4) the creation of “Your Kidneys and Your Health” information booklet as an aid to the review and revision of the concept and content of the nascent resource. The needs assessment was synthesized into a logic model, which outlined the causes of the problem identified and key determinants to target for improvement.

##### Literature Review of Existing Self-management Interventions in CKD

The evidence discussed in the introduction serves as a justification for the focus on self-management in CKD. A further literature review (unpublished) was subsequently carried out to expand on self-management in CKD and identify the existing CKD-specific self-management interventions and ascertain the (1) components of effective self-management interventions for adults with CKD and (2) patient perspectives of CKD self-management interventions.

##### Previous Work Conducted by Our Group

During the early part of phase 1, we conducted a mixed methods cross-sectional observational study (DIMENSION-KD [Investigating lifestyle determinants of muscle and physical function, and the impact on patient experience and support needs in kidney disease]; ISRCTN84422148) investigating the lifestyle determinants of physical function and QoL and their impact on patient experience and support needs in CKD. The study involved several parts, including a survey to identify and evaluate lifestyle determinants and factors associated with living with CKD, semistructured interviews with people living with CKD about their health and experiences of living with a kidney condition, and semistructured interviews with health care professionals (HCPs) about the role of self-management and lifestyle interventions for people living with CKD. To participate in the study, individuals had to be (1) diagnosed with kidney disease, (2) male or female, (3) aged ≥18 years, and (4) willing and able to give informed consent and comply with the study protocol. Individuals were excluded if they (1) were aged <18 years; (2) had any other significant disease or disorder that, in the opinion of the patient’s own clinician, may either put the participants at risk because of participation in the study or may influence the result of the study or the participant’s ability to participate in the study; or (3) had an inability to give informed consent or comply with the protocol for any reason. HCPs were defined as any clinician who cares for patients or professionals on the “Health and Care Professions Council” (eg, physician, general practitioner [GP], practice nurse, dietician, physiotherapist, and occupational therapist).

##### A PPI CKD Priority Workshop

A research priority–setting workshop relating to healthy lifestyle, physical activity, and diet and weight management was conducted to identify topics that were important to people with nondialysis CKD. Participants with stages 3 and 4 nondialysis CKD were invited from the Leicester Kidney Lifestyle Team PPI contact list and included people with CKD who were managed in primary care and their relatives. Participants attended a half-day workshop addressing 3 broad topics: (1) healthy lifestyle, (2) physical activity, and (3) diet and weight management. For each topic, the participants were divided into 2 facilitated discussion groups, and the points arising were then reviewed and evaluated among the whole group to formulate indicative research priority questions. Finally, the participants independently ranked their top three research priorities based on these questions.

##### “Your Kidneys and Your Health” Information Booklet

Moreover, 3 workshops, separate from the priority-setting workshop, were conducted to develop a prototype educational resource to provide a basis for subsequent review and refinement of the content, design, and delivery format. Similar to the priority-setting workshop, participants with stage 3 and 4 nondialysis CKD were invited from the Leicester Kidney Lifestyle Team PPI contact list and included people with CKD who were managed in primary care and their relatives. The workshops were interactive, with brainstorming discussions about content and style. For practical reasons, the initial resource was coproduced in a booklet form to provide a physical document that could be easily examined and refined in real time. The participants annotated the booklet, while graphic designers from the Leicester Biomedical Research Centre updated the document “on the spot” based on the feedback, producing new versions for further comments. Following this process, the booklet was printed, and copies were sent to all PPI members. We also formed a collaborative relationship with a local GP practice who distributed the booklet and collated the feedback. The booklet was developed as an aid to understand the concept and content of the nascent resource and thus was not intended to be the final intervention but to facilitate the early development of the educational program (MK&M).

#### Step 2: Identification of Outcomes, Performance Objectives, and Change Objectives

The next step involved specifying what needed to change in order to achieve the desired outcomes of the intervention. The overall desired outcome was to improve the knowledge, skills, and confidence of people with CKD in managing their health (termed *patient activation*) and consequent self-management behaviors.

The determinants (factors that could be expected to influence behavior) of each desired outcome were identified and relevant performance objectives (factors associated with the expected behaviors required to achieve the program goal) were developed. Combining the determinants and performance objectives, we produced a list of change objectives that refer to what needs to change to affect the performance objective and ultimately the program outcome. For example, for individuals to increase their access to social support and resources, they need to be aware of the support available, understand which support and resources are beneficial to their own personal situation, and have the confidence to access the relevant support and resources. The output of this process is a matrix of change objectives detailing what will be targeted in the intervention. To illustrate the proposed relationships between change methods, the determinants they were expected to influence, and the behavioral and environmental outcomes that would address the problem, we constructed a logic model of change.

#### Step 3: Selection of Theory-Informed Intervention Methods and Practical Strategies

Step 3 included the selection of theoretical methods considered to influence changes in the determinants in the desired direction. This step involved the review of theoretical methods and their translation into practical applications matching the health behavior change of the individual. The criteria for choosing theories to inform interventions, suggested by Hardeman and Sutton [40], were used to help guide the selection of theories. The criteria are as follows: (1) use in interventions aimed at similar target behaviors; (2) applicability to the target group; (3) clear definition of causal, testable pathways between behavioral determinants and behavior; (4) strength of evidence about predictive validity; and (5) clear guidelines for measurement [40]. Appropriate behavior change theories were identified from the literature and expert steering group. These were then translated into strategies for practical application.

#### Step 4: Development of the Intervention Program

The aim of step 4 was to combine the learning from the previous steps into the program and to develop working documents to guide the intervention production [34]. The steering group collaborated with the PPI group to provide guidance on the scope and content of the intervention and the most appropriate format of delivery. Program materials were codeveloped with the PPI group, and feedback was elicited. A description of the scope, content, and delivery of the program was outlined.

### Phase 2: Adoption and Adaptation of the MyDESMOND Platform to Suit MK&M Content and Materials

The MyDESMOND platform interface and features [27] were adopted for MK&M (justification is described in the *Results* section). The developed MK&M materials and content (presented in the *Results* section) were mapped onto the existing MyDESMOND platform [27]. Key components of the intervention were selected based on the feasibility of adopting and adapting the existing MyDESMOND platform to suit MK&M content and materials within the resource constraints.

### Ethics Approval

For the priority-setting workshop and the booklet workshops, the PPI members provided written informed consent. DIMENSION-KD was granted national research ethical approval (18/EM/0117). All patients provided informed written consent, and the study was conducted in accordance with the Declaration of Helsinki. The SMILE-K study was fully approved by the Research Ethics Committee-Leicester South (17/EM/0357). All participants are required to provide informed consent online.

## Results

### Phase 1: Development Process of MK&M Using IM

#### Step 1: Needs Assessment

##### Literature Review

The self-management of a chronic illness or disease involves 3 sets of core tasks, namely, medical, behavioral, and emotional management [41], and encompasses 5 key processes, namely, problem-solving, decision-making, resource utilization, forming partnerships, and action planning [42]. Increased awareness and improved decision-making skills enable patients to be more engaged in the management of their condition [43]. Clinical management guidelines for CKD consider the promotion of self-management behaviors as a standard of care, with recommendations to incorporate information, advice, and education to support self-management behaviors into treatment plans at all stages of CKD [44] to potentially slow the disease progression and prevent complications [45]. Despite recommendations to support CKD self-management, many individuals living with CKD do not perceive the need to self-manage their CKD and lack an understanding of the importance of self-management [17,18]. To help people with CKD self-manage, it is widely recommended that individuals are aware of their diagnosis; involved in shared treatment decisions; provided access to their medical data; and given information on blood pressure control, exercise, diet, medication management, and smoking cessation [46]. However, the unique and complex nature of CKD (ie, asymptomatic and multimorbidity) may require distinct self-management support that may differ from that in other long-term conditions [47].

Previous systematic reviews [48-51] and integrative reviews [7,21,22] of self-management interventions for people living with CKD have shown improvements in self-care activities. However, they are variable in their effectiveness in managing and preventing the progression of CKD. A recent review highlighted a wide range of self-management interventions for adults with CKD (not requiring kidney replacement therapy) with considerable heterogeneity in outcomes [6]. The wide variations in the content, delivery, and components of digital self-management interventions (eg, self-monitoring and education), alongside the outcomes assessed and results obtained, make it difficult to compare findings across studies evaluating self-management programs [52]. Consequently, there is a paucity of knowledge about which components of these programs are the most effective [53].

Despite the importance of theory-driven interventions [54], efforts to improve CKD self-management have been largely atheoretical [22]. Major gaps in the literature include the lack of patient involvement and engagement in intervention design (an estimated <1% of programs are codeveloped with patients) and failure to apply behavior change theory (only 20% are based on a theory or framework) [6]. Person-centeredness, applicability to comorbidities associated with CKD, and physiological and nonphysiological outcomes are also often lacking from self-management interventions [6].

Meaningful information relating to the patient perspective of self-management interventions (eg, desired support and delivery of this support) is lacking from the literature but is key to providing valuable information regarding attitudes towards and challenges associated with self-management and self-management interventions [7]. Furthermore, patients have highlighted that interventions need to be individualized and tailored to their specific situations and preferences (eg, awareness of having CKD, stage of CKD, knowledge of the disease, and access to resources [6]). Engaging patients by involving them in the co-design of such interventions will ensure that their values, culture, and psychosocial needs are addressed in the intervention [7,21,22].

##### Findings From a Mixed Methods Observational Study Exploring Lifestyle Determinants in CKD (DIMENSION-KD)

Our survey findings showed that of 743 people living with nondialysis CKD (mean eGFR 32.3, SD 17.1 mL/min/1.73 m^2^; mean age 67.8, SD 13.9 years; 503/743, 68% male), only a minority of individuals were activated for self-management, with 60% (444/743) reporting low activation (knowledge, skills, and confidence in managing own health) [55]. Patient activation declined concomitantly with disease progression, and low activation was associated with being older, having a comorbidity, and lower hemoglobin levels. Individuals with low activation had poor cardiorespiratory fitness and health-related QoL and more cardiovascular disease risk factors [55].

Findings (unpublished) from semistructured interviews with 22 people living with CKD (mean eGFR 42.2, SD 14.3 mL/min/1.73 m^2^; mean age 71.4, range 48-88 years; 11/22, 50% male) highlighted a need for education and support for people living with CKD (not requiring kidney replacement therapy), particularly during the initial period following their diagnosis. The participants described a desire to understand CKD and its impact on overall health (including disease progression, health risk factors, and comorbidities) and the role self-management and lifestyle can play in living well with CKD. Information about medication, symptoms, lifestyle behaviors (eg, diet and physical activity) and mental health and well-being and strategies to help manage these and make relevant changes were thought to be key to enabling people to manage their CKD. Peer and familial support, goal setting, coping strategies, and self-monitoring tools were considered as motivators to facilitate health and lifestyle changes. In addition, HCPs were interviewed about the role of self-management and lifestyle interventions for people living with CKD. The findings highlight the need to increase patient awareness of CKD and self-management in primary care. There was a belief that individuals need to be empowered to take responsibility to manage their health and engage in healthy lifestyle behaviors (eg, physical activity and weight management).

##### CKD Priority-Setting Workshop Findings

A total of 12 participants (people with CKD: n=9, 75%; and family members: n=3, 25%) attended our priority-setting workshop. The dominant recurring theme was a lack of appropriate information from primary care providers. This not only extended to lifestyle, physical activity, and diet but also to kidney function and CKD itself. Many individuals had little knowledge of the roles of the kidney; the causes and nature of CKD as a condition; or its impact, consequences, and relevance to wider health. Both psychological and social well-being were deemed as important as physical health. Findings highlighted a need to focus on the most appropriate means of disseminating knowledge regarding CKD, lifestyle, diet, and exercise to people living with CKD. The top 3 research priorities were (1) How can we improve GP communication? (2) What can I eat and drink with CKD? and (3) What diet will help me maintain physical activity [56]?

##### Findings From the Development and Evaluation of “Your Kidneys and Your Health” Information Booklet

An education booklet called “Your Kidneys and Your Health” was codeveloped by our team in collaboration with the PPI group (10 people with CKD and 3 family members). Feedback from other people living with CKD, who were given the booklet by their GP, suggested that the most useful aspects were information relating to CKD stages, kidney function, diet, and symptoms associated with CKD. The participants also reported that the content was easy to read and understand. Although the education booklet provided a basic holistic approach to lifestyle management in CKD and represented a convenient, low-cost alternative to delivery of face-to-face education, it was limited in its ability to provide interactive guidance and support and to engage participants in positive behavior change. It was determined that another method (eg, digital) would be required to deliver CKD education and lifestyle information. However, the booklet provided a useful physical resource to share with the PPI and steering groups to aid discussion about the content and design of the larger self-management program.

##### Summary of Needs Assessment Findings

**Figure 1:**
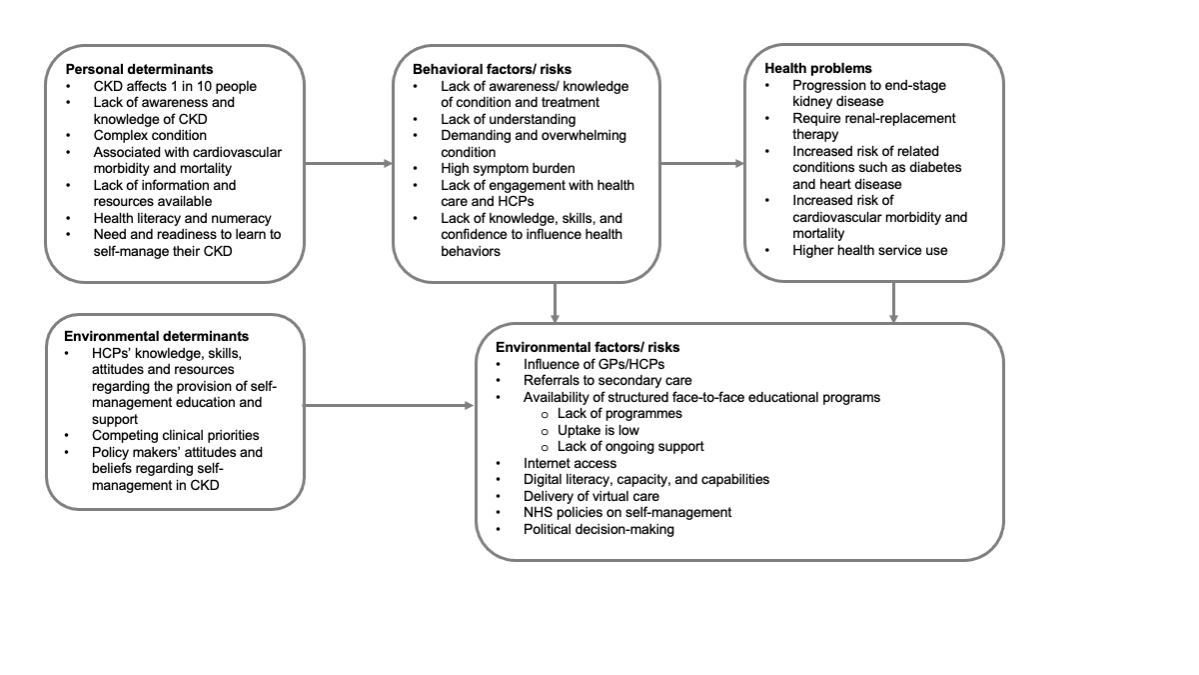
Logic model of problem. CKD: chronic kidney disease; HCP: health care professional; NHS: National Health Service; GP: general practitioner.

The findings from the different elements of the needs assessment, alongside learning from previous interventions and resources, were collated to establish a rationale for a self-management intervention for people with CKD. The needs assessment highlighted that individuals with CKD have multiple needs that can differ based on the complexity of their illness, health knowledge, and confidence to manage the disease. Having explored the issues of CKD self-management, a logic model was created to better understand the problem, which illustrates in detail the issues under investigation and the associated relationships and factors (Figure 1). It was agreed that the goal was to co-design (meaningful stakeholder involvement in the planning and development of the intervention to ensure that it meets their needs and is usable [57,58]) a self-management intervention that could be offered to any individual with nondialysis CKD. On the basis of the evidence discussed in the introduction, needs assessment, and experience of the steering group, it was determined that a potential self-management program would be best delivered digitally to enable accessible, tailored, and interactive information and support and to overcome the barriers experienced in the delivery of PACT, SPARK, and the “Your Kidneys and Your Health” booklet. The PPI steering group named the proposed self-management program MK&M.

#### Step 2: Identification of Outcomes, Performance Objectives, and Change Objectives

In step 2, the findings from step 1 were used to specify what would need to be changed for people to successfully self-manage their CKD. The findings from the needs assessment indicated that people with CKD would benefit from education about the condition and lifestyle behaviors as early as possible following diagnosis. In addition to containing information to improve knowledge, the intervention should also aim to improve and maintain self-management behaviors, promote self-care skills, increase self-efficacy, and improve well-being. Thus, the program goals were to improve the knowledge, skills, and confidence of people with CKD in managing their health and consequent self-management behaviors. The key outcomes were to (1) increase patient activation (ie, knowledge, skills, and confidence); (2) reduce health risks; (3) manage symptoms; and (4) increase physical function.

**Figure 2:**
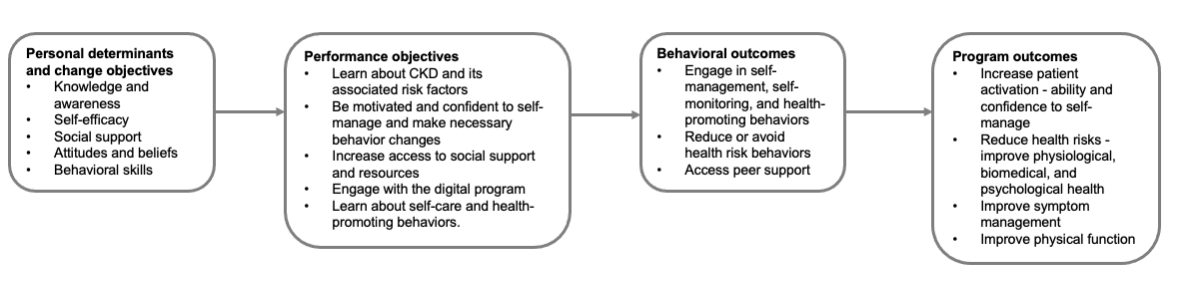
Logic model of change. CKD: chronic kidney disease.

The behavioral determinants were (1) knowledge and awareness, (2) self-efficacy, (3) social support, (4) attitudes and beliefs (intention), and (5) behavioral skills. The performance objectives were (1) learn about CKD and its associated risk factors, (2) be motivated and confident to self-manage and make necessary behavior changes, (3) increase access to social support and resources, (4) engage with the digital program, and (5) learn about self-care and health-promoting behaviors. A matrix of change objectives detailing the behavioral determinants required to influence the performance objectives, and subsequently self-management behavior, in people with CKD was created. The change objectives required to achieve the performance objectives are detailed in Table 1. A logic model of change illustrating the proposed relationships between change methods, the determinants they were expected to influence, and the behavioral and environmental outcomes that would address the problem are presented in Figure 2.

**Table 1:** Change objectives.

Performance objectives	Personal determinants					External determinants
	Knowledge and awareness	Self-efficacy	Attitudes and beliefs (intention)	Behavioral skills	Social support	
Learn about CKD^a^ and its associated risk factors	Awareness and understanding about CKD, its treatment options, and its symptoms and associated health risks and conditions	Demonstrates ability to process information about CKD and self-management Expresses confidence in applying the information learnt	Recognizes that CKD is a long-term condition that requires patients to take an active role in looking after their health and reducing health risks	Be able to identify own personal health risk factors and methods to modify and/or reduce health risk factors and problems	N/A^b^	
Be motivated and confident to self-manage and make necessary behavior changes	Understand the importance of self-managing and identify which factors and behaviors can be modified	Be motivated and confident to self-manage and make necessary behavior changes	Belief that there will be benefits in making necessary behavior changes to their health and lifestyle	Develop routines that assist in managing their health Develop habits that enhance self-management	N/A	
Increase access to social support and resources	Increase awareness of the support available and knowledge of which support and resources are beneficial to own personal situation	Have the confidence to access the relevant support and resources	N/A	N/A	Accesses and receives support from both HCPs^c^ and others with CKD	
Willingness to engage with the program and accept more responsibility for managing their health and health care	Learn about the importance and benefits of looking after their health and health care	Express confidence in managing their health and health care Express confidence in taking action to help prevent or minimize symptoms or health problems	Increase the motivation to use the program Expresses willingness to take greater responsibility toward managing their CKD, health, and health care	Be able to find a solution when new situations or health problems arise Be able to confidently recognize own limitations and seek necessary help when required	Engage with other program users	
Learn about and practice self-care and health-promoting behaviors	Awareness and understanding of health-promoting behaviors	Express confidence in self-managing and performing health-promoting behaviors	Recognizes and believes that practicing self-management behaviors will enhance health and well-being Expected to reduce health risks and engage in health-promoting behaviors	Be able to confidently perform and maintain self-care practices and health-promoting behaviors, including lifestyle changes Demonstrates model behaviors	N/A	

^a^CKD: chronic kidney disease.

^b^N/A: not applicable.

^c^HCP: health care professional.

#### Step 3: Selection of Theory-Informed Intervention Methods and Practical Strategies

In developing MK&M, we were able to draw upon multiple theories that consider the complexity of behavior change, which are commonly used in the development of health behavior change interventions and have been extensively used in chronic disease self-management interventions [59], including those designed to improve self-management behaviors, and thus provide an effective framework for self-management support in people with CKD [60]. The theories and frameworks selected (self-management framework; Capability, Opportunity, Motivation Behavior model components of Behaviour Change Wheel and taxonomy of behavior change techniques; health action process approach model; common sense model; and social cognitive theory) are described in detail, including the justification for inclusion, in Multimedia Appendix 1 [61-80]. A theoretical framework was constructed to map the relevant mechanisms of action (processes that influence behaviors), behavior change techniques, and application of practical strategies to facilitate behavior change in MK&M and is illustrated in Table 2.

**Table 2:** My Kidneys & Me theoretical framework, target determinants, mechanisms of action (MoA), behavior change techniques (BCTs), and practical applications.

Guiding principle	Underlying theory and constructs	Target determinants^a^	MoA	BCTs	Methods and practical applications
Provide patients with education about CKD^b^ and promote self-management	COM-B^c^: psychological capability HAPA^d^: risk awareness and outcome expectancies CSM^e^: interpretation of the problem SCT^f^: outcome expectations, behavioral capability, and self-regulation	Environment: address the perceived lack of support in providing clear disease-based information	Knowledge	Information about the condition, including causes, symptoms, treatment, and consequences Biofeedback Feedback on behavior	Educational material, including videos, providing information about the kidneys; chronic nature of CKD; possible causes and consequences of CKD; conditions associated with CKD; health risks; medication; health care appointments; types of treatments; symptoms; and healthy lifestyle, including diet and physical activity Interactive quizzes to test knowledge and learning “How to” sessions proving guidance on how to perform self-care and health-promoting behaviors and strategies to encourage behavior change and the adoption of new behaviors Trackers to monitor health and activity and to provide feedback on behavior outcomes
Provide patients with education about CKD^b^ and promote self-management	COM- B: psychological capability HAPA: risk awareness and outcome expectancies CSM: interpretation of the problem SCT: outcome expectancy, behavioral capability, and self-regulation	Behavioral: address outcome expectations (risks and benefits) of engaging in self-management behaviors and other healthy lifestyles	Behavioral regulation	Information on the consequences of behavior Instruction on how to perform behavior Modeling or demonstrating behavior Focus on past success Feedback on behavior Biofeedback Self-monitoring of behavior	Educational material, including videos, providing information about health risks associated with CKD and the concept of personal responsibility for health focusing on modifiable CVD^g^ risk factors Interactive quizzes to test knowledge and learning “How to” sessions encouraging users to engage with the self-monitoring trackers and to complete an action plan for desired behaviors Trackers (health trackers, symptom tracker, and exercise trackers [step count and strength training]) are available for users to record their physical activity, including monitoring steps, strength training, weight, dietary intake, and symptoms
Provide guidance and opportunity to self-monitor health and self-management behaviors	COM-B: physical capability HAPA: risk awareness, outcome expectancies, self-efficacy, planning, and action control CSM: action plan, coping and appraisal SCT: outcome expectancy, behavioral capability, self-efficacy, and self-regulation	Environmental: address the lack of support in providing information regarding self-management and healthy lifestyle behaviors Behavioral: address the benefits of engaging in health-promoting behaviors and how they can help to support symptom management, for example, fatigue, and help individuals stay independent, re-enforcing innate desires and outcome expectancies for self-management Personal: Guidelines and advice on what is safe and appropriate may help to reduce fears related to recognizing performance limitations	Skill	Information on desired behaviors Instruction on how to perform behavior Behavioral rehearsal/ practice Coping strategies Habit formation	Educational material provides information about healthy lifestyle, physical activity, and well-being. Information on benefits and consequences of behavior is highlighted “How to” sessions provide information on how to perform health-promoting behaviors and helpful strategies to encourage adoption and maintenance of health-promoting behaviors. Instructions on how to set SMART^h^ goals and create personalized action plans Trackers can encourage users to track and self-monitor their health, symptoms, and exercise behaviors Chat forum enables users to share their experiences and strategies with others
Provide advice about how to self-manage through a healthy lifestyle, goal setting (set health and behavior-related goals), self-monitoring health, and self-management behaviors	COM-B: reflective motivation HAPA: outcome expectancies, self-efficacy, planning, and action control CSM: action plan, coping and appraisal SCT: self-efficacy, behavior capability, observational learning, and self-regulation	Behavioral: address outcome expectations (risks and benefits) of engaging in self-management and health-promoting behaviors	Beliefs about capabilities	Information and examples of desired behaviors Verbal persuasion to encourage the adoption of new behaviors Verbal persuasion to reduce self-doubts and enhance self-efficacy Focus on past successes Habit formation	Videos highlighting important messages are presented by experts to encourage users to set and review personalized goals, create and continue their action plans, and engage in changes of health and lifestyle behaviors Educational material addressing knowledge about health-promoting behaviors, factors related to health concerns, and physiological responses, including those to exercise Chat forum enables verbal reassurance and encouragement among users that may increase their self-efficacy
Provide advice about how to self-manage through a healthy lifestyle, goal setting (set health and behavior-related goals), self-monitoring health, and self-management behaviors	COM-B: reflective motivation HAPA: outcome expectancies, self-efficacy, planning, and action control CSM: action plan, coping and appraisal SCT: self-efficacy, behavior capability, observational learning, and self-regulation	Environmental: the lack of guidance on how to set health- and behavior-related goals Behavioral: Target self-management and health motivations by including self-regulation strategies Personal: Target the innate drives for self-management by asking participants to consider why self-management and health-promoting behaviors may be important to them and how it would specifically benefit them in their lives	Motivations and goals	Information on the consequences of behaviors Information on goal setting, reviewing goals, action planning, problem-solving, self-monitoring	Educational material, including videos, provides information and guidance on how to set personalized goals, review goals, create action plans, and evaluate progress. Information was also provided on barrier identification and how to address/ overcome potential barriers Users are encouraged to set goals and complete an action plan for target behaviors, considering any potential barriers and strategies to overcome these Access to goal-setting functions enables users to set health-related and exercise goals
Provide advice about how to self-manage through a healthy lifestyle, goal setting (set health and behavior-related goals), self-monitoring health, and self-management behaviors	COM-B: reflective motivation HAPA: outcome expectancies, self-efficacy, planning, and action control CSM: action plan, coping and appraisal SCT: self-efficacy, behavior capability, observational learning, and self-regulation	Behavioral: address the perceptions of living with CKD, expectations of self-managing the condition, and importance of engaging in health-promoting behaviors	Role and identity	Information on desired behaviors Verbal persuasion to encourage the adoption of new behaviors Message framing/reframing	Videos presented by experts provide key information and important messages about CKD, associated health risks, symptoms, health care appointments, physical activity and exercise, diet, well-being, sleep, and other relevant information
Provide advice about how to manage the emotional consequences of CKD and the opportunity to share experiences	COM-B: autonomic motivation SCT: outcome expectancy, collective efficacy, and self-regulation	Environment: addresses the need for emotional support Personal: enable participants to share their experiences of living with CKD	Emotions	Information on appropriate support and resources Reduce negative emotions Coping strategies Social support Social comparison	Educational material provides information around well-being, including how to cope and live with CKD. Videos are presented by experts on managing emotions and accessing support Chat forum encourages users to talk to others, including HCPs, family and friends, and other people with CKD. It also enables users to provide and receive peer support
Provide the opportunity to share positive self-management experiences and advice from peers	COM-B: social opportunity SCT: outcome expectancy and collective efficacy	Environment: addresses the need for peer support and facilitate peer learning Personal: enable participants to share positive experiences of engaging in self-care and health-promoting behaviors and seek advice from peers	Social influences	Social support Social comparison Encouragement	Chat forum enables users to discuss and share their thoughts and experiences, providing encouragement for each other. It also enables users to seek and provide approval from others

^a^Target determinants include personal, behavioral, and environmental influences identified from previous work to target within the intervention.

^b^CKD: chronic kidney disease.

^c^COM-B: Capability, Opportunity, Motivation Behavior model.

^d^HAPA: health action process approach model.

^e^CSM: common sense model.

^f^SCT: social cognitive theory.

^g^CVD: cardiovascular disease.

^h^SMART: Smart, Measurable, Achievable, Realistic, Timely.

#### Step 4: Development of the Intervention Program

##### Defining the Content

The overall requirements of MK&M were derived from the needs assessment and theory described earlier. Discussions with stakeholders, including people living with CKD, and findings from qualitative interviews and PPI workshops highlighted an interest in digital interactive information, including videos, as a way to understand CKD self-management. We codeveloped content and material that was designed to improve kidney-specific self-management practices, including increasing CKD knowledge, reducing health risks, managing symptoms, and improving physical function. The content was reviewed and refined by a wide range of CKD experts representing the kidney multidisciplinary team, including specialist kidney clinicians, nurses, dieticians, pharmacists, physiotherapists, and psychologists, and the PPI group. Educational “Learn About” sessions were developed to provide information about kidneys; CKD; its treatment, symptoms, and associated health risks; and lifestyle-related factors (eg, diet and physical activity [including strength training]). Behavior change–focused “How To” sessions were designed to provide practical strategies to assist individuals with making small modifications to improve their health and lifestyle behaviors. The “How To” sessions also contained information about how to set goals, self-monitor health and behaviors, and create action plans, as goal setting is a motivator to facilitate health and lifestyle behavior changes. Moreover, enabling individuals to set, review, and adapt their own goals is important [49,81]; therefore, goal-setting features were included to support people in selecting and self-monitoring their own goals. Good physical function is important for patients with CKD [82] but is often overlooked and underemphasized in exercise guidance. Therefore, based on the findings from the SPARK evaluation and feedback, we developed a separate strength training educational session with strength exercise programs, videos demonstrating the exercises, and a bespoke strength tracker.

The development of the content for MK&M was an iterative process, with 12 PPI members providing regular review and input, such as suggesting (1) a symptom tracker to enable self-monitoring of symptoms; (2) the inclusion of new sessions on sleep and well-being, emphasizing the importance of looking after mental health, including coping and living with CKD; (3) the use of “myth and fact” quizzes as a way to test knowledge; and (4) stating how long sessions should take to complete. These suggestions resulted in the following actions: (1) designing a bespoke symptom tracker, (2) creating both educational and “how to” sessions on improving sleep quality and looking after well-being, (3) adding quizzes to both “learn about” and “how to” sessions, and (4) including the anticipated time it would take people to work through and complete each session (based on the mean time of completion for PPI group). Content topics included are detailed in Multimedia Appendix 2.

##### Defining the Format of Delivery

Although the content and materials of MK&M were developed with a future digital platform in mind (based on previous learning, needs assessment, and PPI feedback), before this point, the exact web platform had not yet been decided. Following discussions about the design and format of the web platform for MK&M, it was agreed among the steering group that the MyDESMOND platform represented an ideal existing platform (reasons detailed in the subsequent section) to host MK&M; therefore, the final stage of development involved the required adaptation of the MyDESMOND interface and features [27] for MK&M content and materials.

### Phase 2: Adoption and Adaptation of the Existing MyDESMOND Platform to Suit MK&M Content and Materials

Given the successful development and implementation of the MyDESMOND program [27,83,84], it was advantageous to use the preexisting and established MyDESMOND platform to host the MK&M program. MyDESMOND is an award-winning program on a quality-assured platform, which is known to be well accepted and effective [83]. The MyDESMOND platform was built using progressive web apps and is accessible on any digital device (tablet, desktop computer, and smartphone) and operating system. The system was developed using the Zend framework and MySQL database. The app includes an app programming interface built with Zend, which is used to manage data between the AngularJS progressive web app and the database. The MyDESMOND platform hosts several evidence-based web-based self-management programs, including programs for people at risk of or with type 2 diabetes. The existing MyDESMOND programs are used throughout the United Kingdom and Australia. These MyDESMOND programs have gained accreditation from Quality Institute for Self-Management Education and Training. The Quality Institute for Self-Management Education and Training (QISMET) defines good practice in self-management education [85]. MyDESMOND has been successfully reviewed by Organisation for the Review of Care and Health Apps (ORCHA) [86], an independent leading digital health reviewer that assesses digital health programs against the latest standards and guidelines that cover clinical/ professional assurance, data and privacy, and usability and accessibility. MyDESMOND also conforms to the Digital Technology Assessment Criteria (DTAC) for health and social care. This is a new national baseline criterion used to ensure that the latest standards within digital health systems are being met. It covers clinical safety, data protection, technical security, interoperability, and usability and accessibility standards and is used for the commissioning of digital health technologies [87]. The MyDESMOND platform is flexible to easily allow for additional programs to be added and adapted. Adding the MK&M content and materials to the MyDESMOND platform [27] and adapting the existing platform to suit the program needs provided a cost- and time-efficient approach for developing our digital intervention. In addition, the MyDESMOND platform meets many National Health Service (NHS) requirements and quality standards, which would enable the future implementation of MK&M in clinical practice.

**Figure 3:**
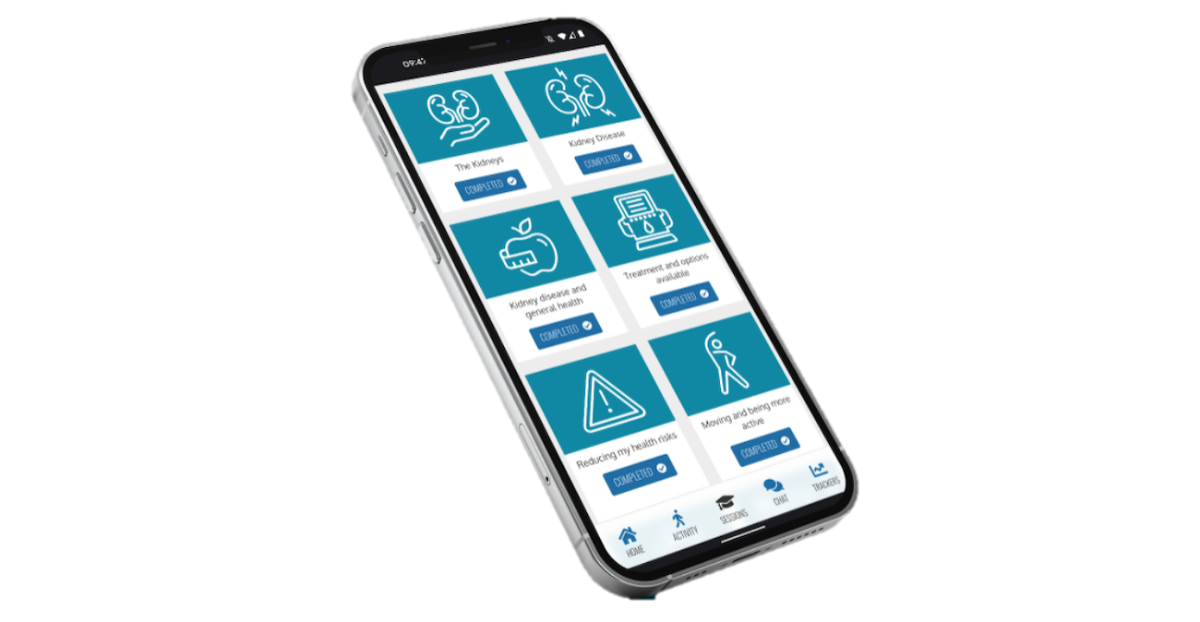
Example of learning sessions available on My Kidneys & Me.

**Figure 4:**
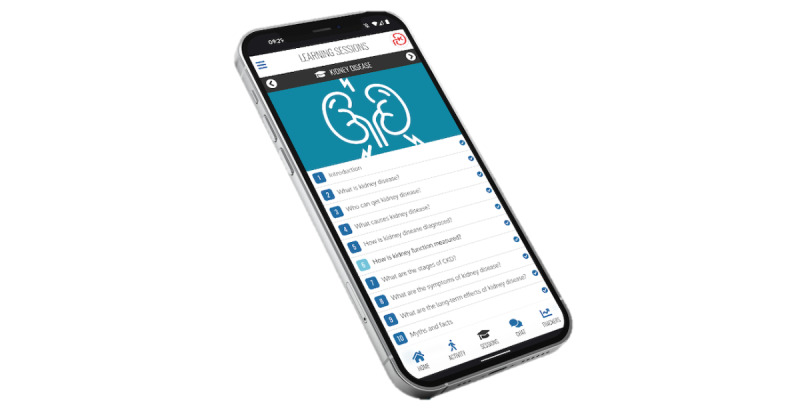
Example of the “Kidney Disease” learning sessions available on My Kidneys & Me.

We mapped the developed MK&M content as closely as possible to the preexisting features already available on the MyDESMOND platform [27]. For example, we mapped the “Learn About” and “How To” sessions onto the preexisting “Learning” and “Booster” sessions on the platform. Figures 3 and 4 present examples of the sessions available. The MK&M goal-setting materials were mapped onto the MyDESMOND “Decision Maker” feature. We adopted the health (eg, blood pressure, shape, and healthy eating) and physical activity (eg, step counting) trackers that were already available on the MyDESMOND platform [27], as these were perceived to adequately meet the needs of people with CKD.

**Figure 5:**
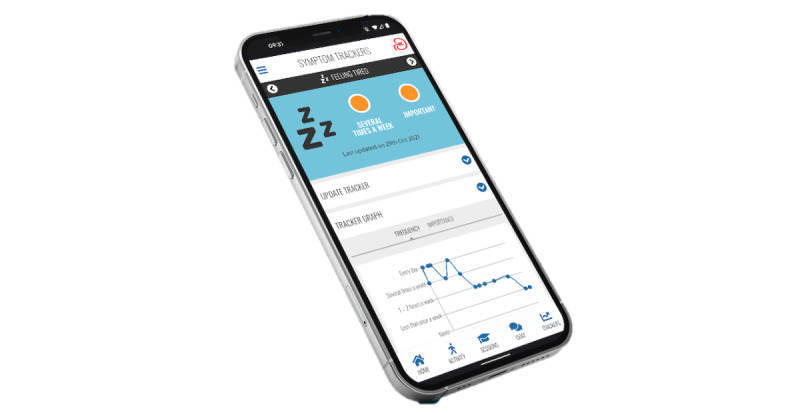
Chronic kidney disease symptom tracker on My Kidneys & Me.

**Figure 6:**
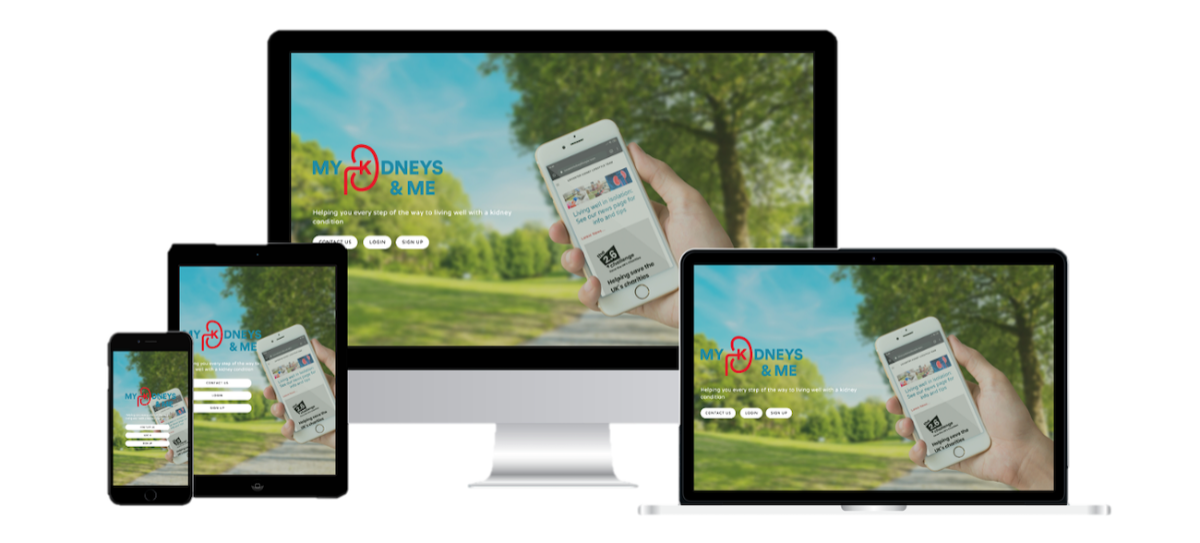
Example of My Kidneys & Me launch page and the digital devices it is accessible on.

Although we were able to adopt the interface and features of the MyDESMOND platform [27] and map most of the MK&M content and materials onto it, there were some key MK&M components that were missing. Thus, we further adapted the platform to suit our needs. This involved creating and realizing a bespoke CKD symptom (detailing 13 common symptoms identified by people with CKD not receiving kidney replacement therapy [88]) (Figure 5) and a bespoke strength tracker, which were conceptualized during the codevelopment of materials and content in phase 1. Links to strength training materials, including flashcards and demonstration videos, were embedded within MK&M. To aid individuals’ understanding of the program and navigation through the platform, we also created a welcome video detailing the aims of the program and a screencast (ie, digital video recording of our computer screen, including audio narration) explaining how to navigate the program and the key features available. The finalized MK&M materials and content were uploaded onto the platform by the MyDESMOND digital team. Finally, we added an MK&M-specific launch page to the MyDESMOND platform, as shown in Figure 6. Usability (functionality, navigation, and interactivity) and experience testing of MK&M was conducted through think-aloud interviews to identify potential areas for refinement (manuscript in preparation).

## Discussion

### Principal Findings

Self-management has been identified as a top priority by individuals with nondialysis CKD, their caregivers, clinicians, and policy makers [89]. The concept of patient empowerment is integrated in the “NHS Long Term Plan,” which aims to support people with long-term conditions to improve self-management skills and provide access to resources [90]. Here, we have described the codevelopment of a theory- and evidence-based digital self-management program for people with CKD, guided by the IM framework and the MyDESMOND development process [27].

### Steering Group

The formation, participation, and engagement of the multidisciplinary planning group and PPI group were valuable as it enabled knowledge, experiences, and visions to be shared. The development process was guided by the inputs from HCPs and the PPI group, with both groups reviewing and refining the program content and materials and the method of delivery alongside the researchers. This ensured that the program was appropriately designed and relevant to people with CKD and that people with CKD and HCPs support MK&M. Having input from the MyDESMOND and IMPACT teams greatly facilitated the development of MK&M, as learning first-hand from their experiences in developing MyDESMOND [27], and adapting their processes, was invaluable. In addition, the ability to map our own program onto the MyDESMOND platform was advantageous.

### Applying Theoretical Framework

The IM process helped the rigorous development of MK&M and ensured that we explored our program goals in depth and were able to specify what it was that we needed to change and which method and practical applications would elicit the desired change. This ensured that MK&M, similar to MyDESMOND, was underpinned by a strong theoretical framework and evidence-based foundation. Using IM enabled a meaningful analysis of the underlying mechanisms that are hypothesized to affect the desired intervention outcomes by enabling the explicit linking of interventional components to theory, which should result in improved outcomes for the target population [91]. The use of behavior change theories and techniques to determine the anticipated behavior change ensured a pragmatic application of change methods.

### Practical Implications

The IM framework provides a comprehensive approach that clearly articulates the development process [34], which allows for more transparency in the design of the intervention to others outside the development team. The process may be different for different interventions, and each step of the framework allows for the adaptation of the different requirements of the specific population, setting, and nature of the intervention [34]. Using IM enabled us to produce an intervention that is potentially efficacious and acceptable and can be implemented in CKD. The needs assessment conducted in step 1, alongside lessons learnt from our previous work, provided us with a clear understanding of the different key components that needed to be incorporated into MK&M. Identifying the desired outcomes of MK&M ensured that appropriate content and materials were designed to address our program goals.

Aside from using IM to develop interventions, the approach may aid clinicians in assessing the suitability of an intervention, its applicability to their setting and population, or what change may need to occur to the intervention to enhance its suitability [92]. The dissemination of the development of complex interventions offers clinicians more detailed information on which they can base their decision regarding the implementation of these interventions [92].

### Comparison With Other Interventions

To our knowledge, this is the first systematically developed theory- and evidence-based digital self-management program for people with nondialysis CKD in the United Kingdom. Another digital intervention for people with CKD, called Kidney Beam, has been developed in the United Kingdom to provide support for exercise, physical activity, and emotional well-being [93]. Similar to MK&M, Kidney Beam is theory informed and guided by the Behaviour Change Wheel. The full development process, application of theory, and selection of BCTs have yet to be published. Unlike MK&M, which provides a holistic approach to self-management, encompassing the core tasks alongside the key processes, Kidney Beam aims to specifically address exercise, physical activity, and emotional well-being. Results of the feasibility study were promising and showed good levels of engagement, with 43% of individuals signing up to the platform and completing at least one exercise class [93]. Similar to MK&M, Kidney Beam is currently being evaluated in a randomized controlled trial to evaluate the feasibility, clinical value, and cost-effectiveness of the Kidney Beam program delivered as part of clinical care [93]. It is to be hoped that MK&M and Kidney Beam will provide complementary options for future kidney care.

Digital self-management programs for people with CKD have also been developed in other countries such as Canada [94] and the United States [95]. There are similarities and differences between these programs. Like us, Donald et al [94] and Markossian et al [95] co-designed their program with stakeholders, involving people living with CKD and their relatives, clinicians, researchers, and graphic designers. Although there are similarities in methodologies, both MK&M and “My Kidneys My Health” [94] developed a web-based self-management resource, whereas Markossian et al [95] developed a mobile app. In our program, we used the IM framework to guide the development, whereas Donald et al [94] used the “Knowledge-to-Action” framework to guide the multiphased activities for determining CKD patient self-management support intervention. It is not clear what framework was used to guide the development of the mobile app by Markossian et al [95]. Like us, Donald et al [94] and Markossian et al [95] are evaluating their programs in a study, which will further provide an insight into the acceptability and engagement of the digital programs and identification of patient-reported outcomes and potential factors for implementation.

### Evaluation and Implementation

The efficacy of MK&M in improving patient activation and subsequent self-management behaviors is currently being evaluated in a multisite clinical trial in the United Kingdom, with results expected in 2023 (protocol paper published elsewhere [96]). Patient activation is considered the cornerstone of effective self-management in CKD, as it encompasses all the necessary ingredients for effective self-management (ie, knowledge, skills, and confidence) [45]. Findings from the trial will provide the first step in determining whether MK&M is effective in increasing patient activation and promoting self-management behaviors in people with nondialysis CKD. In addition to evaluating MK&M, we also look to implement MK&M in renal services as a part of clinical care. Currently renal services are undergoing redesigning as a part of the Renal Service Transformation Programme [97]; consequently, we are conducting preliminary work before implementing MK&M into clinical care to ensure MK&M can be successfully embedded within the new model of care.

### Strengths and Limitations

The inclusion of a stakeholder steering group comprising multidisciplinary professionals and PPI members with a variety of experience and expertise, who were involved in the codevelopment of MK&M, was vital to the successful development of MK&M. The PPI group was key in identifying the needs and preferences of people with CKD; however, the PPI members were predominantly White British, female, and well educated. Consequently, as our PPI group may not be representative of the CKD population, MK&M may not be suitable for all individuals with CKD. Successful use of MK&M is likely to require a certain level of health literacy and activation. It was developed in partnership with individuals who participate and engage in activities such as PPI and are therefore likely to be more engaged than the typical patient. The generalizability and applicability of MK&M will be established and addressed during the evaluation, refinement, and implementation phases of the program.

Although the IM approach provides a useful planning template to develop a tool that is theoretically grounded and evidence-based, it is very time consuming. The process described in this paper took approximately 2 years to complete, which was longer than initially anticipated; this was partly because of the COVID-19 pandemic, where members of the participatory steering group had competing clinical interests that took priority during this period, and the time taken to adapt a preexisting platform to suit our needs. Despite this, others have also reported similar experiences when using IM [36,98,99].

Despite the ambition, and initially setting out, to coproduce MK&M, it was not feasible owing to the COVID-19 pandemic. The inability to meet face-to-face during this period influenced the level of involvement of the members of the participatory steering group; moreover, clinical members were required to work on the frontline of the United Kingdom’s NHS, and people with kidney disease were required to shield themselves because of being classed as clinically vulnerable. Although the steering group actively inputted during this time, it was not to the degree that we initially envisaged when we began this work; however, we did what we were able to during such challenging circumstances. Thus, we consider this work to be co-designed with the elements of coproduction.

### Future Suggestions

We acknowledge that MK&M may not meet the needs of those with lower levels of activation and/or health literacy. The findings from our mixed methods evaluation of MK&M will provide a wealth of information, including details regarding program usage, engagement, and adherence. Exploring patients’ and HCPs’ views and experiences of using MK&M will provide an in-depth understanding of its usability, functionality, and acceptability. The findings will inform the potential improvements and adaptations we could make to the program to ensure that it is more acceptable and accessible to disadvantaged groups. In addition, the program requires digital literacy and thus may exclude individuals who are not computer literate or do not have access to digital technology. More work is needed to improve digital literacy and ensure the provision of internet access. There is also scope for digital programs such as MK&M to address the health inequalities that exist in the CKD population, and further work is needed to explore how these programs could effectively reduce health inequalities in CKD.

### Conclusions

This paper provides a detailed example of the application of IM in the development of a theory- and evidence-based digital self-management program for people living with CKD. We have been successful in developing a digital holistic CKD-specific self-management program that provides accessible, tailored, and interactive information and support to help people with CKD improve their awareness and understanding of their condition, health knowledge, and confidence in managing their condition. MK&M aims to provide ongoing self-management education, support, and guidance for people living with CKD to manage their condition and live full, productive lives. Results from the multicenter randomized controlled trial evaluating MK&M will provide evidence of the efficacy, effectiveness, and cost-effectiveness of the program. If MK&M is successful in increasing patient activation and subsequent self-management behaviors, we anticipate that it will also improve people’s QoL, physical function, and symptom burden while reducing the number of hospital admissions and saving costs to the NHS.

## Appendix

Multimedia Appendix 1 Description of theories and frameworks selected and the rationale.

Multimedia Appendix 2

## Abbreviations

BCT: behavior change technique
CKD: chronic kidney disease
DESMOND: Diabetes Education and Self-Management for Ongoing and Newly Diagnosed
DHI: digital health intervention
DIMENSION-KD: Investigating lifestyle determinants of muscle and physical function, and the impact on patient experience and support needs in kidney disease
GP: general practitioner
HCP: health care professional
IM: intervention mapping
MK&M: My Kidneys & Me
NHS: National Health Service
PACT: Physical Activity Changing Together
PPI: patient and public involvement
QoL: quality of life
SMILE-K: Self-Management Intervention through Lifestyle Education for Kidney health
SPARK: Self-directed Programme to Increase Physical Activity in Chronic Kidney Disease

## References

[1] Kim LG, Cleary F, Wheeler DC, Caplin B, Nitsch D, Hull SA, UK National Chronic Kidney Disease Audit (2018). How do primary care doctors in England and Wales code and manage people with chronic kidney disease? Results from the National Chronic Kidney Disease Audit. Nephrol Dial Transplant.

[2] Webster AC, Nagler EV, Morton RL, Masson P (2017). Chronic kidney disease. Lancet.

[3] Go AS, Chertow GM, Fan D, McCulloch CE, Hsu C (2004). Chronic kidney disease and the risks of death, cardiovascular events, and hospitalization. N Engl J Med.

[4] Kalantar-Zadeh K, Jafar TH, Nitsch D, Neuen BL, Perkovic V (2021). Chronic kidney disease. Lancet.

[5] Neale EP, Middleton J, Lambert K (2020). Barriers and enablers to detection and management of chronic kidney disease in primary healthcare: a systematic review. BMC Nephrol.

[6] Donald M, Kahlon BK, Beanlands H, Straus S, Ronksley P, Herrington G, Tong A, Grill A, Waldvogel B, Large CA, Large CL, Harwood L, Novak M, James MT, Elliott M, Fernandez N, Brimble S, Samuel S, Hemmelgarn BR (2018). Self-management interventions for adults with chronic kidney disease: a scoping review. BMJ Open.

[7] Havas K, Bonner A, Douglas C (2016). Self-management support for people with chronic kidney disease: patient perspectives. J Ren Care.

[8] Wagner EH (1998). Chronic disease management: what will it take to improve care for chronic illness?. Eff Clin Pract.

[9] Davies MJ, Heller S, Skinner TC, Campbell MJ, Carey ME, Cradock S, Dallosso HM, Daly H, Doherty Y, Eaton S, Fox C, Oliver L, Rantell K, Rayman G, Khunti K, Diabetes Education Self Management for Ongoing Newly Diagnosed Collaborative (2008). Effectiveness of the diabetes education and self management for ongoing and newly diagnosed (DESMOND) programme for people with newly diagnosed type 2 diabetes: cluster randomised controlled trial. BMJ.

[10] Novak M, Costantini L, Schneider S, Beanlands H (2013). Approaches to self-management in chronic illness. Semin Dial.

[11] Schulman-Green D, Jaser S, Martin F, Alonzo A, Grey M, McCorkle R, Redeker NS, Reynolds N, Whittemore R (2012). Processes of self-management in chronic illness. J Nurs Scholarsh.

[12] Peng S, He J, Huang J, Lun L, Zeng J, Zeng S, Zhang L, Liu X, Wu Y (2019). Self-management interventions for chronic kidney disease: a systematic review and meta-analysis. BMC Nephrol.

[13] Hibbard JH, Mahoney ER, Stock R, Tusler M (2007). Do increases in patient activation result in improved self-management behaviors?. Health Serv Res.

[14] Welch JL, Bartlett Ellis RJ, Perkins SM, Johnson CS, Zimmerman LM, Russell CL, Richards C, Guise DM, Decker BS (2016). Knowledge and awareness among patients with chronic kidney disease stage 3. Nephrol Nurs J.

[15] Molnar AO, Akbari A, Brimble KS (2020). Perceived and objective kidney disease knowledge in patients with advanced CKD followed in a multidisciplinary CKD clinic. Can J Kidney Health Dis.

[16] Finkelstein FO, Story K, Firanek C, Barre P, Takano T, Soroka S, Mujais S, Rodd K, Mendelssohn D (2008). Perceived knowledge among patients cared for by nephrologists about chronic kidney disease and end-stage renal disease therapies. Kidney Int.

[17] Narva AS, Norton JM, Boulware LE (2015). Educating patients about CKD: the path to self-management and patient-centered care. Clin J Am Soc Nephrology.

[18] Greer RC, Crews DC, Boulware LE (2012). Challenges perceived by primary care providers to educating patients about chronic kidney disease. J Ren Care.

[19] Greer RC, Cooper LA, Crews DC, Powe NR, Boulware LE (2011). Quality of patient-physician discussions about CKD in primary care: a cross-sectional study. Am J Kidney Dis.

[20] Lee YJ, Shin SJ, Wang RH, Lin KD, Lee YL, Wang YH (2016). Pathways of empowerment perceptions, health literacy, self-efficacy, and self-care behaviors to glycemic control in patients with type 2 diabetes mellitus. Patient Educ Couns.

[21] Welch JL, Johnson M, Zimmerman L, Russell CL, Perkins SM, Decker BS (2015). Self-management interventions in stages 1 to 4 chronic kidney disease: an integrative review. West J Nurs Res.

[22] Bonner A, Havas K, Douglas C, Thepha T, Bennett P, Clark R (2014). Self-management programmes in stages 1-4 chronic kidney disease: a literature review. J Ren Care.

[23] Murray E, Burns J, See TS, Lai R, Nazareth I (2005). Interactive Health Communication Applications for people with chronic disease. Cochrane Database Syst Rev.

[24] Slater H, Campbell JM, Stinson JN, Burley MM, Briggs AM (2017). End user and implementer experiences of mHealth technologies for noncommunicable chronic disease management in young adults: systematic review. J Med Internet Res.

[25] Slater H, Dear BF, Merolli MA, Li LC, Briggs AM (2016). Use of eHealth technologies to enable the implementation of musculoskeletal Models of Care: evidence and practice. Best Pract Res Clin Rheumatol.

[26] Craig P, Dieppe P, Macintyre S, Michie S, Nazareth I, Petticrew M, Medical Research Council Guidance (2008). Developing and evaluating complex interventions: the new Medical Research Council guidance. BMJ.

[27] Hadjiconstantinou M, Schreder S, Brough C, Northern A, Stribling B, Khunti K, Davies MJ (2020). Using intervention mapping to develop a digital self-management program for people with type 2 diabetes: tutorial on MyDESMOND. J Med Internet Res.

[28] Bandura A (1986). Social Foundations of Thought and Action: A Social Cognitive Theory.

[29] Clarke A, MacKinnon H, Yates T, Smith A (2016). Mp412 The “person-based approach” to developing a structured group education programme to increase physical activity in CKD: the pact-project. Nephrology Dialysis Transplantation.

[30] Clarke A (2018). Developing behaviour change interventions to increase levels of physical activity in patients with chronic kidney disease. University of Leicester.

[31] Ajzen I (1991). The theory of planned behavior. Organizational Behav Human Decision Processes.

[32] MacKinnon H, Clarke A, Singh S, Smith A (2016). MP409 Development of a self-directed programme to increase physical activity in chronic kidney disease. Nephrology Dialysis Transplantation.

[33] Mackinnon H (2022).

[34] Eldredge LK, Markham CM, Ruiter RA, Fernández ME, Kok G, Parcel GS (2016). Planning Health Promotion Programs: An Intervention Mapping Approach, 4th Edition.

[35] Kok G, Peters LW, Ruiter RA (2017). Planning theory- and evidence-based behavior change interventions: a conceptual review of the intervention mapping protocol. Psicol Reflex Crit.

[36] Detaille SI, van der Gulden JW, Engels JA, Heerkens YF, van Dijk FJH (2010). Using intervention mapping (IM) to develop a self-management programme for employees with a chronic disease in the Netherlands. BMC Public Health.

[37] Geense WW, van Gaal BG, Knoll JL, Cornelissen EA, Schoonhoven L, Kok G (2016). Online support program for parents of children with a chronic kidney disease using intervention mapping: a development and evaluation protocol. JMIR Res Protoc.

[38] Shen H, van der Kleij R, van der Boog PJ, Song X, Wang W, Zhang T, Li Z, Lou X, Chavannes N (2020). Development and evaluation of an eHealth self-management intervention for patients with chronic kidney disease in China: protocol for a mixed-method hybrid type 2 trial. BMC Nephrol.

[39] Been-Dahmen JM, Beck DK, Peeters MA, van der Stege H, Tielen M, van Buren MC, Ista E, van Staa A, Massey EK (2019). Evaluating the feasibility of a nurse-led self-management support intervention for kidney transplant recipients: a pilot study. BMC Nephrol.

[40] Hardeman W, Sutton S, Griffin S, Johnston M, White A, Wareham NJ, Kinmonth AL (2005). A causal modelling approach to the development of theory-based behaviour change programmes for trial evaluation. Health Educ Res.

[41] Corbin J, Strauss A (1985). Managing chronic illness at home: three lines of work. Qual Sociol.

[42] Lorig KR, Holman H (2003). Self-management education: history, definition, outcomes, and mechanisms. Ann Behav Med.

[43] Morton K, Dennison L, May C, Murray E, Little P, McManus R, Yardley L (2017). Using digital interventions for self-management of chronic physical health conditions: a meta-ethnography review of published studies. Patient Educ Couns.

[44] Kidney Disease: Improving Global Outcomes (KDIGO) CKD-MBD Update Work Group (2017). KDIGO 2017 Clinical Practice Guideline Update for the diagnosis, evaluation, prevention, and treatment of Chronic Kidney Disease-Mineral and Bone Disorder (CKD-MBD). Kidney Int Suppl.

[45] Lightfoot CJ, Nair D, Bennett PN, Smith AC, Griffin AD, Warren M, Wilkinson TJ (2022). Patient activation: the cornerstone of effective self-management in chronic kidney disease?. Kidney Dialysis.

[46] (2021). Chronic Kidney Disease: Assessment and Management.

[47] Donald M, Beanlands H, Straus S, Ronksley P, Tam-Tham H, Finlay J, MacKay J, Elliott M, Herrington G, Harwood L, Large CA, Large CL, Waldvogel B, Sparkes D, Delgado M, Tong A, Grill A, Novak M, James MT, Brimble KS, Samuel S, Hemmelgarn BR (2019). Identifying needs for self-management interventions for adults with CKD and their caregivers: a qualitative study. Am J Kidney Dis.

[48] Lin MY, Liu MF, Hsu LF, Tsai PS (2017). Effects of self-management on chronic kidney disease: a meta-analysis. Int J Nurs Stud.

[49] Lopez-Vargas PA, Tong A, Howell M, Craig JC (2016). Educational interventions for patients with CKD: a systematic review. Am J Kidney Dis.

[50] Lee MC, Wu SV, Hsieh NC, Tsai JM (2016). Self-management programs on eGFR, depression, and quality of life among patients with chronic kidney disease: a meta-analysis. Asian Nurs Res (Korean Soc Nurs Sci).

[51] Mason J, Khunti K, Stone M, Farooqi A, Carr S (2008). Educational interventions in kidney disease care: a systematic review of randomized trials. Am J Kidney Dis.

[52] Shen H, van der Kleij RM, van der Boog PJ, Chang X, Chavannes NH (2019). Electronic health self-management interventions for patients with chronic kidney disease: systematic review of quantitative and qualitative evidence. J Med Internet Res.

[53] Zimbudzi E, Lo C, Misso ML, Ranasinha S, Kerr PG, Teede HJ, Zoungas S (2018). Effectiveness of self-management support interventions for people with comorbid diabetes and chronic kidney disease: a systematic review and meta-analysis. Syst Rev.

[54] Glanz K, Bishop DB (2010). The role of behavioral science theory in development and implementation of public health interventions. Annu Rev Public Health.

[55] Wilkinson TJ, Memory K, Lightfoot CJ, Palmer J, Smith AC (2021). Determinants of patient activation and its association with cardiovascular disease risk in chronic kidney disease: a cross-sectional study. Health Expect.

[56] Wilkinson T, Goodliffe S, Wilde L, Smith A P176 -“I’ve been diagnosed since 1970 and nobody has ever given me advice on diet or lifestyle”: a research priority setting workshop with CKD patients treated in primary care. University Of Leicester.

[57] Vargas C, Whelan J, Brimblecombe J, Allender S (2022). Co-creation, co-design, co-production for public health - a perspective on definition and distinctions. Public Health Res Pract.

[58] Clarke D, Jones F, Harris R, Robert G, Collaborative Rehabilitation Environments in Acute Stroke (CREATE) team (2017). What outcomes are associated with developing and implementing co-produced interventions in acute healthcare settings? A rapid evidence synthesis. BMJ Open.

[59] Allegrante JP, Wells MT, Peterson JC (2019). Interventions to support behavioral self-management of chronic diseases. Annu Rev Public Health.

[60] Havas K, Douglas C, Bonner A (2018). Meeting patients where they are: improving outcomes in early chronic kidney disease with tailored self-management support (the CKD-SMS study). BMC Nephrol.

[61] Corbin JM, Strauss A (1988). Unending Work and Care: Managing Chronic Illness at Home.

[62] Atkins L, Francis J, Islam R, O'Connor D, Patey A, Ivers N, Foy R, Duncan EM, Colquhoun H, Grimshaw JM, Lawton R, Michie S (2017). A guide to using the Theoretical Domains Framework of behaviour change to investigate implementation problems. Implement Sci.

[63] Michie S, Johnston M, Francis J, Hardeman W, Eccles M (2008). From theory to intervention: mapping theoretically derived behavioural determinants to behaviour change techniques. Applied Psychol.

[64] Michie S, van Stralen MM, West R (2011). The behaviour change wheel: a new method for characterising and designing behaviour change interventions. Implement Sci.

[65] Michie S, Atkins L, West R (2014). The Behaviour Change Wheel: A Guide to Designing Interventions.

[66] Schwarzer R (1992). Self-efficacy in the adoption and maintenance of health behaviors: theoretical approaches and a new model. Self-efficacy: Thought Control of Action.

[67] MacPhail M, Mullan B, Sharpe L, MacCann C, Todd J (2014). Using the health action process approach to predict and improve health outcomes in individuals with type 2 diabetes mellitus. Diabetes Metab Syndr Obes.

[68] Bandura A (1997). Self-efficacy: The Exercise of Control.

[69] Leventhal H, Meyer D, Nerenz D (1980). The common sense model of illness danger. Medical Psychology.

[70] Leventhal H, Phillips LA, Burns E (2016). The Common-Sense Model of Self-Regulation (CSM): a dynamic framework for understanding illness self-management. J Behav Med.

[71] Hale ED, Treharne GJ, Kitas GD (2007). The common-sense model of self-regulation of health and illness: how can we use it to understand and respond to our patients' needs?. Rheumatology (Oxford).

[72] Pagels AA, Söderquist BK, Heiwe S (2015). Differences in illness representations in patients with chronic kidney disease. J Ren Care.

[73] Wu C, Lin C, Hsieh H, Chang S (2016). Lived experiences and illness representation of Taiwanese patients with late-stage chronic kidney disease. J Health Psychol.

[74] Lin CC, Chen MC, Hsieh HF, Chang SC (2013). Illness representations and coping processes of Taiwanese patients with early-stage chronic kidney disease. J Nurs Res.

[75] Schwarzer R, Fuchs R (2010). Changing risk behaviors and adopting health behaviors: the role of self-efficacy beliefs. Self-efficacy in Changing Societies.

[76] Wu SV, Courtney M, Edwards H, McDowell J, Shortridge-Baggett LM, Chang P (2007). Self-efficacy, outcome expectations and self-care behaviour in people with type 2 diabetes in Taiwan. J Clin Nurs.

[77] Bandura A (2004). Health promotion by social cognitive means. Health Educ Behav.

[78] Curtin RB, Walters BA, Schatell D, Pennell P, Wise M, Klicko K (2008). Self-efficacy and self-management behaviors in patients with chronic kidney disease. Adv Chronic Kidney Dis.

[79] Michie S, Abraham C, Whittington C, McAteer J, Gupta S (2009). Effective techniques in healthy eating and physical activity interventions: a meta-regression. Health Psychol.

[80] Clarke AL, Young HM, Hull KL, Hudson N, Burton JO, Smith AC (2015). Motivations and barriers to exercise in chronic kidney disease: a qualitative study. Nephrol Dial Transplant.

[81] Evangelidis N, Craig J, Bauman A, Manera K, Saglimbene V, Tong A (2019). Lifestyle behaviour change for preventing the progression of chronic kidney disease: a systematic review. BMJ Open.

[82] MacKinnon HJ, Wilkinson TJ, Clarke AL, Gould DW, O'Sullivan TF, Xenophontos S, Watson EL, Singh SJ, Smith AC (2018). The association of physical function and physical activity with all-cause mortality and adverse clinical outcomes in nondialysis chronic kidney disease: a systematic review. Ther Adv Chronic Dis.

[83] Quinn LM, Davies MJ, Northern A, Brough C, Schreder S, Stribling B, Khunti K, Hadjiconstantinou M (2021). Use of MyDesmond digital education programme to support self-management in people with type 2 diabetes during the COVID-19 pandemic. Diabet Med.

[84] Hadjiconstantinou M, Barker MM, Brough C, Schreder S, Northern A, Stribling B, Khunti K, Davies MJ (2021). Improved diabetes-related distress and self-efficacy outcomes in a self-management digital programme for people with type 2 diabetes, myDESMOND. Diabet Med.

[85] Qismet.

[86] ORCHA Health.

[87] Digital Technology Assessment Criteria. National Health Service.

[88] Brown SA, Tyrer F, Clarke AL, Lloyd-Davies LH, Niyi-Odumosu FA, Nah RG, Stein AG, Tarrant C, Smith AC (2018). Kidney symptom questionnaire: development, content validation and relationship with quality of life. J Ren Care.

[89] Hemmelgarn BR, Pannu N, Ahmed SB, Elliott MJ, Tam-Tham H, Lillie E, Straus SE, Donald M, Barnieh L, Chong GC, Hillier DR, Huffman KT, Lei AC, Villanueva BV, Young DM, Fowler EA, Manns BJ, Laupacis A (2017). Determining the research priorities for patients with chronic kidney disease not on dialysis. Nephrol Dial Transplant.

[90] NHS Long Term Plan. National Health Service.

[91] French SD, Green SE, O'Connor DA, McKenzie JE, Francis JJ, Michie S, Buchbinder R, Schattner P, Spike N, Grimshaw JM (2012). Developing theory-informed behaviour change interventions to implement evidence into practice: a systematic approach using the Theoretical Domains Framework. Implement Sci.

[92] Jones TM, Dear BF, Hush JM, Titov N, Dean CM (2016). Application of intervention mapping to the development of a complex physical therapist intervention. Phys Ther.

[93] Mayes J, Billany RE, Vadaszy N, Young HM, Castle EM, Bishop NC, Bramham K, Nixon AC, Wilkinson TJ, Hamilton AJ, Saynor ZL, Chilcot J, Picariello F, Macdonald J, Greenwood SA (2022). The rapid development of a novel kidney-specific digital intervention for self-management of physical activity and emotional well-being during the COVID-19 pandemic and beyond: kidney beam. Clin Kidney J.

[94] Donald M, Beanlands H, Straus SE, Smekal M, Gil S, Elliott MJ, Herrington G, Harwood L, Waldvogel B, Delgado M, Sparkes D, Tong A, Grill A, Novak M, James MT, Brimble KS, Samuel S, Tu K, Farragher J, Hemmelgarn BR (2021). A web-based self-management support prototype for adults with chronic kidney disease (my kidneys my health): co-design and usability testing. JMIR Form Res.

[95] Markossian TW, Boyda J, Taylor J, Etingen B, Modave F, Price R, Kramer HJ (2021). A mobile app to support self-management of chronic kidney disease: development study. JMIR Hum Factors.

[96] Lightfoot CJ, Wilkinson TJ, Yates T, Davies MJ, Smith AC (2022). ‘Self-Management Intervention through Lifestyle Education for Kidney health’ (the SMILE-K study): protocol for a single-blind longitudinal randomised control trial with nested pilot study. medRxiv.

[97] Jenkins K (2021). J Kidney Care.

[98] McEachan RR, Lawton RJ, Jackson C, Conner M, Lunt J (2008). Evidence, theory and context: using intervention mapping to develop a worksite physical activity intervention. BMC Public Health.

[99] Munir F, Kalawsky K, Wallis DJ, Donaldson-Feilder E (2013). Using intervention mapping to develop a work-related guidance tool for those affected by cancer. BMC Public Health.

