# Supplementary material for: The Codevelopment of “My Kidneys & Me”: A Digital Self-management Program for People With Chronic Kidney Disease
Source: J Med Internet Res. 2022 Nov 14;24(11):e39657. doi: 10.2196/39657 (PMC9706383; doi:10.2196/39657)
Supplement: Multimedia Appendix 1 [file jmir_v24i11e39657_app1.docx]

**Supplementary Material 1**

**Description of theories and frameworks selected and the rationale**

**Self-Management Framework**

The Corbin and Strauss’ Self-Management Framework[63] proposes that self-management of a chronic condition comprises three distinct sets of activities: (1) medical management of the condition, e.g. taking medication, (2) behavioural management, e.g. adopting new behaviours in the context of a chronic disease, and (3) emotional management, e.g. dealing with the feelings of frustration, fright, and despair associated with chronic disease. As the focus is often on the medical management of conditions, we wanted to include all three elements into the MK&M programme. In addition, we wanted to integrate the processes of self-management, proposed by Lorig, Holman [42] as these are perceived to be the core skills involved in self-management. These skills include problem solving, decision making, utilising resources, partnering with healthcare providers, taking action, and improving self-efficacy.

**COM-B and Taxonomy of BCTs**

The COM-B model was applied as interventions which utilise behaviour change techniques (BCTs) are acknowledged as effective in influencing changes in health behaviours[64-66]. The focus was mainly on the COM-B components as the Behaviour Change Wheel (BCW) determined these as the three essential conditions for behaviour[67]. The method for designing behaviour change interventions using the BCW outlined by Michie et al. [68] was utilised. This step-by-step process involves understanding the behaviour, identifying intervention options, and identifying content and implementation options. To guide this process, the APEASE (Affordability, Practicality, Effectiveness and cost-effectiveness, Acceptability, Side-effects/safety, and Equity) criteria[68] was utilised.

**Health Action Process Approach Model**

The Health Action Process Approach (HAPA) model can explain health behaviour engagement[69], and suggests that the adoption, initiation, and maintenance of health behaviour consists of two processes: forming an intention (goal setting) followed by a stage of planning to act and action (goal pursuit). Risk awareness (perceived health threat) and outcome expectancies (belief that behaviour will bring about desired change) lead to the formation of an intention to either adopt health protective behaviours or change health risk behaviours[70] and are predisposing factors to goal setting. Planning and action control lead to the adoption and maintenance of health behaviours, and are influential in goal pursuit. Perceived self-efficacy plays a crucial role at all stages[71]; if an individual does not believe in their ability to perform desired actions, that individual will fail to adopt, initiate and maintain the behaviour. Thus, we aimed to incorporate the HAPA constructs and processes into the development of MK&M: goal setting (including risk awareness and outcome expectancies), goal pursuit (including planning and action control), and self-efficacy.

**Common Sense Model**

The Common Sense Model (CSM) for Health and Illness[72] provides a conceptual framework for understanding the perceptual, cognitive, and behavioural processes involved in the self-management of health threats including adherence to treatments and lifestyle changes[73]. It proposes that individuals are “active problem seekers” who will undertake behaviours that are consistent with reducing health threats. Illness perceptions (organised patterns of thought generated in response to a threat) may inform an individual’s coping strategies including their willingness to engage in self-management behaviours. The CSM suggests individuals perceive an illness based on the following principles: 1) identity (the name and symptoms that go with it); 2) cause (the perceived cause of the condition); 3) timeline (whether they believe it to be chronic or not); 4) consequences (how the illness will impact on them physically and socially); and 5) curability/controllability (the degree to which the illness can be cured or managed and the role the individual plays in making this happen)[74]. In addition to the cognitive appraisal of symptoms and illness, individuals form parallel emotional responses to health threats such as feelings of fear or distress[72]. People with earlier stages of CKD or with fewer symptoms do not perceive their condition as being a threat, and do not always relate their symptoms to CKD[75]. Patients with CKD who hold a better understanding of the relationship between the condition and lifestyle factors (illness coherence) have been shown to describe stronger intentions to adopt a healthier lifestyle[76]. Individuals who adopt effective coping strategies can reduce the health threat; changing lifestyle can provide a sense of personal control in reducing the rate of illness progression for individuals with CKD[77]. Thus, it was important to include perceptions of CKD risk and management, and strategies to adopt lifestyle changes.

**Social Cognitive Theory**

Social Cognitive Theory[28] explains how people regulate their behaviour through control and reinforcement to achieve goal-directed behaviour that can be maintained over time. SCT synthesizes concepts and processes from cognitive, behaviouristic, and emotional models of behaviour change, so it can be readily applied to interventions for disease prevention and management. One of the main constructs of the theory is self-efficacy, which is an important prerequisite for health-related behaviour change[78]; it is considered the most important variable for the prediction of self-care behaviours in people with chronic diseases[79]. The SCT aims to promote self-management behaviours, including adoption of healthy lifestyle, through self-regulating cognitive processes by improving knowledge, self-efficacy, and problem-solving skills[80]. Higher perceived self-efficacy in individuals with CKD has been found to be associated with increased self-management behaviours including communication, partnership, self-care, and medication-adherence behaviours[81]. The other constructs include knowledge (only as a pre-condition to change), outcome expectancies, goal setting and facilitators, and impediments to change. Goal-setting and self-monitoring are useful components of effective interventions[82], and have been identified as key motivators for health behaviour (e.g. exercise) interventions by people with CKD[83]; thus, it was important to incorporate these components into MK&M.
