# Supplementary material for: The Codevelopment of “My Kidneys & Me”: A Digital Self-management Program for People With Chronic Kidney Disease
Source: J Med Internet Res. 2022 Nov 14;24(11):e39657. doi: 10.2196/39657 (PMC9706383; doi:10.2196/39657)
Supplement: Multimedia Appendix 2 [file jmir_v24i11e39657_app2.docx]

**Supplementary Material 2**

**Summary of MK&M educational material**

Each topic has a learning session (education material covering key information) and a booster session (information about how to engage in health-promoting behaviours).

| Topic | Summary of sessions |
| --- | --- |
| 1. The kidneys | This is an introduction about the kidneys, including what the kidneys are, what they do, why they are important, and what happens when they are not working properly. |
| 1. Kidney disease | This session looks at what kidney disease is, who can have it, potential causes, how it is diagnosed, how kidney function is measured, the signs and symptoms, and the long-term effects. |
| 1. Kidney disease and general health | This session looks at how kidney disease may impact overall health, potential health risk factors and other health problems. |
| 1. Treatment options available | This session covers information about healthcare team involvement, healthcare appointments, medications, dialysis and transplantation. |
| 1. Reducing my health risks | This session covers the importance of self-management, reducing health risk factors, and management of other health conditions. |
| 1. Moving more and being active | This session covers the current recommendations for adults, the importance of physical activity and its role in the management of kidney disease, and increasing/ maintaining activity levels. |
| 1. Keeping my muscles healthy | This session focuses on the importance of muscle health. It will cover what happens to muscles as you get older, why keeping muscles healthy is important for those living with kidney disease, and what you can do to help your muscles stay as strong as possible including how to safely take part in strength training exercises. |
| 1. Eating a healthy balanced diet | This session looks at kidney health in relation to food choices and adaptation of recipes. |
| 1. Managing my symptoms | This session covers symptom management and looks at the most commonly reported symptoms, monitoring symptoms, and strategies to help manage symptoms. |
| 1. Improving my sleep quality | This session focuses on the importance of good sleep quality, potential sleep problems, monitoring sleep, and improving sleep quality. |
| 1. Looking after my well-being | This session looks at the importance of mental health, coping with living with kidney disease, managing illness related stress, and the importance of social support. |
| 1. Goal setting | This session introduces patients to SMART goals, importance of goals, setting goals, and creating and using an action plan. |
